# Medication beliefs among patients with inflammatory bowel disease who report low quality of life: a qualitative study

**DOI:** 10.1186/1471-230X-7-20

**Published:** 2007-06-08

**Authors:** Nicola J Hall, Gregory P Rubin, APS Hungin, Audrey Dougall

**Affiliations:** 1 School of Health, Natural and Social Sciences, University of Sunderland, Sunderland, UK; 2 Centre for Integrated Health Care Research, Durham University, Stockton on Tees, UK; 3 Department of Anthropology, Durham University, Stockton on Tees, UK

## Abstract

**Background:**

Non-adherence to drug therapy is common in Inflammatory Bowel Disease (IBD). Patients' beliefs about treatment have an important influence on adherence. An in-depth understanding of this area is, therefore, important for patient-centred care. The aim of the study was to assess patients' perspectives and beliefs about their medication and to determine how this relates to medicine taking and other related health behaviour as part of a larger qualitative study on health care related behaviour in patients with IBD.

**Methods:**

Individual semi-structured interviews and focus groups. An iterative approach following principles of grounded theory was applied to data collection and analysis.

**Results:**

Main emerging themes were: balance of perceived necessity versus concerns, perceived impact of symptoms and willingness to self-manage medication. There was a clear distinction made between steroids and other preparations. Concerns included the fear of both short and long-term side-effects (mainly steroids), uncertainties about drug interactions and development of long-term immunity. Adapting to and accepting medication use was linked to acceptance of IBD.

**Conclusion:**

A concordant approach including flexible and pro-active support as well as accurate information is important in assisting patients with IBD to self-manage their medication effectively. Health professionals should be aware that attitudes to medicine taking and other related behaviours may be medicine specific and change over time.

## Background

Inflammatory Bowel Disease (IBD) is a chronic disorder characterised by periods of remission and unpredictable relapses. Although there is currently no cure, medical management of the disease includes long-term drug therapy which can be effective in inducing or maintaining remission. Non-adherence to long-term medical therapy is common in chronic disease and typically tends to be around 30–40% [1,2]. Differences in measurement, definition and context, however, can influence reported adherence rates, correlates and outcomes [3]. Reported non-adherence rates in IBD range from 5% to 60% [3,5-8]. Known influential factors on adherence in IBD include socio-demographic characteristics, dosing, depression, disease awareness, the therapeutic relationship, disease activity and psychosocial characteristics [1,5-11]. Within the wider literature on adherence, there is evidence to suggest that patients' treatment and illness beliefs have an important influence on their decisions about treatment [1,9,12-15] and that many people have fairly negative or ambivalent views of medicines [16,17]. Little, however, is known about the attitudes and beliefs that IBD patients have regarding their medication despite evidence of considerable concerns over medication side-effects within this group of patients [18]. Furthermore, research suggests that patients are reluctant to discuss concerns about medication with their doctor [19]. An in-depth understanding of patient treatment beliefs is therefore important for patient-centred care in IBD. We undertook a qualitative analysis of patients' beliefs about drug treatment and how this affected their use of medications as part of a larger study on IBD patients' beliefs and attitudes towards their condition and health care seeking behaviour. The analysis of the latter study centred around the core concept of "health-related normality" and is reported elsewhere [20]. This paper presents a further in-depth analysis of the issues surrounding "medicine taking" and related health behaviours which were important elements within the narratives of the respondents.

## Methods

### Design and participants

58 individuals with established IBD, defined as more than two years since diagnosis, were invited to participate. All those invited had scored in the lowest quartile of a disease specific health-related quality of life measure, the UK-IBDQ [21], in a previous study of a community population from the North East of England [22]. It was anticipated that these individuals would have a wide range of experience of relapse and medicine taking due to their low health-related quality of life scores. 31 individuals participated in the research (19 female and 12 male). 17 had a diagnosis of ulcerative colitis and 14 crohn's disease. 15 took part in individual interviews (10 female and 5 male, mean age 48.3 years, range 26 years to 68 years) and a further 16 in three focus groups (1 female group (n = 7, mean age 46.6 years, range 37 years to 61 years), 1 male group (n = 6, mean age 49.6 years, range 36 years to 72 years) and 1 mixed group (n = 3, mean age 77.4 years, range 76 years to 79 years)). Participants were predominantly from an urban setting but showed diversity in terms of their socio-demographic characteristics and included patients not under specialist care.

Because detailed instructions of the methods employed have been published elsewhere [20], only a brief summary is presented here. Individual interviews were conducted at the respondent's home by either NH or AD. Focus groups were facilitated by both NH and AD. The interview agenda covered individuals' perspectives of their experiences and understandings of their illness, health care seeking behaviour and medication. A flexible approach was employed which allowed emergent themes to be incorporated into later interviews following principles of grounded theory [23]. All interviews and focus groups were tape recorded and transcribed verbatim with the participants' written consent. Interviews continued on a theoretical sampling basis until saturation of themes was reached. Approval was obtained from North Tees Local Ethics Committee.

### Analysis

Concurrent data collection and analysis was undertaken based on principles of grounded theory. The computer software QSR NUD.IST was used to aid indexing and charting. Open and axial coding was carried out independently by NH and AD. Where discrepancies existed these were discussed with a third researcher until a consensus was reached. Examples of open codes included: benefits of medication, side-effects and supply of medication at home. Throughout the analysis, accounts, informants and categories were compared for differences and similarities between them using a constant comparative approach [24]. Data collection continued until no new categories could be found i.e. saturation had been achieved. Respondent validation was obtained by asking respondents to indicate on a five point Likert scale whether initial findings reflected a true representation of their opinion or that of the group (97% either agreed or strongly agreed). Participants were also given the opportunity to add further comments and these were incorporated into the ongoing analysis. The core category of "health-related normality" has been described elsewhere [20]. This paper is based on a further in-depth description of the emerging category "medication", which was found to be an important element within the narratives of the respondents during the analysis and was closely linked to the core category. This analysis was completed following the writing and re-writing process [25] and complements the previously published paper. Particular attention was paid to re-reading the original transcripts and the use of the constant comparison method to ensure that the analysis remained grounded in the narratives of the respondents.

## Results

**Figure 1:**
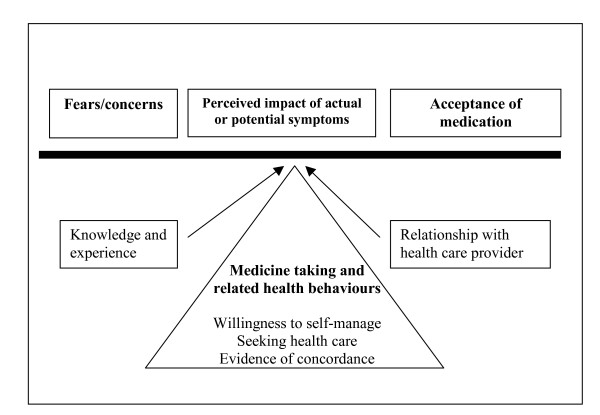
Model of relationship between categories.

Participants offered rich in-depth narratives of their experiences with medication, particularly in regards to side-effects. In general, there was a clear knowledge about the names, dosages and uses of their medications including those for other medical conditions. Medication was an important aspect of the participants' lives and they took advantage of the focus groups to share their experiences and reflect upon these in an open way. They showed interest in the medication of other participants and used the group to normalise their own experiences. Participants had both positive and negative conflicting attitudes towards their medication. The main emerging themes centred on their acceptance and perceived necessity of their medication, the fears and concerns held towards their medication, the perceived impact, actual or potential, that their illness and symptoms had on their lives and willingness to self-manage. The ways in which patients used and self-managed their medicines were determined by an ever-changing balance between their beliefs about their medication and the impact of the illness. The perceived need for pain relief or corticosteroid treatment, for example, can be an important crisis trigger for seeking health care when the necessity of the medication outweighs the barriers to seeking care or the concerns about side-effects, particularly when the perceived impact of the symptoms is felt to be high. Figure 1 illustrates how the category of medicine taking and other associated health behaviours relates to the beliefs about the acceptance and perceived necessity of medication, the balance of fears and concerns and the perceived impact of symptoms. Experience of the illness, knowledge and the relationship with the health care provider can also be influential in this process. This is followed by a more in-depth explanation of the elements within the model.

### Acceptance and perceived necessity of medication

For the majority of participants, medication was seen as necessary in controlling symptoms or flare-ups despite being seen as a nuisance, concerns for side-effects (mainly steroids) and anti-drug feelings. Acceptance of the necessity to take medication was something that was seen to happen over time and was linked to acceptance of the illness (see table 1).

**Table 1:** Acceptance of medication taking

*"At the beginning it was very hard to come to terms with. You feel like you are pill-popping ... but now I have come to terms with the illness and it (taking tablets) is something that I live with. I just have to." *(female with ulcerative colitis, 36 years)

*"It's not a very nice feeling actually to know that you're going to be dependent on tablets for the rest of your life, especially when you're not keen on taking tablets.. I mean I know I have to and I will be taking them for the rest of my days, so you just have to accept it don't you?" *(female with crohn's disease, 44 years)

Despite general anti-drug feelings, taking medication for IBD had became a normal part of everyday life in the same way that living with the consequences of IBD symptoms had. Moreover, it also allowed the maintenance of what was considered to be a "normal life" in that controlling the symptoms with medication meant decreasing the impact of actual or potential distressing symptoms and embarrassment associated with the illness. *"It (taking medication) is something that I do everyday now. So it is nothing out of the ordinary really*." (female with ulcerative colitis, 36 years). Nonetheless, for some participants, due to the nature of the disease, being "ill" was sometimes seen as a periodical rather than a continual state and referred to times of flare-up. In these instances, medication taking only occurred during times of "illness". *"I only take it (medication) when I need to take it. I wouldn't like to be permanently on medication I suppose, only if I had a really good reason to be.., I mean I will take it when I am ill*..." (male with ulcerative colitis, 38 years). Similarly, a participant in the male focus group described his feelings regarding colifoam enemas, which he had been instructed to take regardless of whether he was feeling well. More importance was placed on his negative feelings towards the treatment during remission when the perceived necessity decreased. "*In the end I got sick of doing it because it's not natural to want to,.. I am not being crude but sticking something like that up your backside regardless if you are well or not." *(male with ulcerative colitis, 54 years).

Individual perceptions of the effects of medications were important in determining perceived necessity. For instance, a few participants described how they had "*tested the water*" by coming off maintenance medication. Worsening symptoms strengthened their beliefs about the necessity of continued usage. Those participants unable to identify such a direct link reported more inconsistency in their medication taking (see table 2).

**Table 2:** Testing the water

*"about 2 years ago I tried not taking it (5 ASA therapy) to see what would happen and it did make the condition worse, so I went straight back on it and I take it now*." (male with ulcerative colitis, 40 years)

"*I don't like taking the amount of tablets they were giving me and what I was finding was that even when I was taking the tablets for 6 months I would still have flare-up. I could knock off the tablets for 6 months and have nothing so there didn't seem to be any cause and effect... I would take them for a couple of weeks and then I would lapse again*." (female with ulcerative colitis, 42 years)

Although there was awareness of the need to monitor the bowel for the development of cancer, for those patients with ulcerative colitis on 5-ASA therapy, the prophylactic effect of their maintenance medication against colorectal cancer was not mentioned in either the focus groups or the interviews. Due to ethical considerations, being aware of this particular benefit of the medication was not asked as a direct question to groups or during interviews as the researchers did not wish to instil concerns of cancer risk in individuals who were not already aware.

### Medication fears and concerns

There was a clear distinction made between steroids and other preparations. Steroids were viewed a lot more negatively than the other "*regular*" medication taken for IBD and the fear of dependency was greater. Participants talked about both long and short-term side-effects. *"It's (taking medication) a fact of life... It is something you have to do to make life bearable; it doesn't bother me at all... They (steroids) bother me... I wouldn't like to be on steroids long-term." *(female with ulcerative colitis, 65 years). *"That's what's given me the osteoporosis." *(male with crohn's disease, 31 years). In general the short-term effects were non-gender specific and verbalised as: *"I got mood swings", "it just wasn't me", "you feel euphoric", "I could have eaten the bricks on the wall"*. Fear of being prescribed steroids could in some cases result in a delay in seeking health care. Experience of the effects of the treatment could, nevertheless, counteract some of the negative attitudes. One woman with ulcerative colitis, for example, described her fear of dependency on steroids after a serious flare-up when her "*normal*" medication "*had stopped working*". She had been surprised that her course of steroids had only lasted one week before an improvement in symptoms was evident and this experience had alleviated her fear of further courses, despite her continuing concerns about side-effects (see table 3).

**Table 3:** Steroidal preparations

*"I have been told that if I get any bad flare-ups the next course of treatment after this Asacol is steroids and I have seen what steroids can do to different people. I have a friend on this steroidal thing for his chest and he's got a big jowl and it's just one of these things I am tempted to put off*." (male with ulcerative colitis, 55 years).

*" I didn't know it was only going to be a week and luckily things did resolve but I thought at the start that this was the start of me being dependent on them and I was concerned about that...I was so relieved when the symptoms subsided you know after a week*." (female with ulcerative colitis, 56 years).

*" but the steroids I must say they really did the trick. Big time." *(male with ulcerative colitis 36 years)

*"I was quite amazed at how fast the steroids started to work*." (female with crohn's disease, 44 years)

Negative attitudes to drugs, therefore, seemed to be related to general anti-drug feelings and to specific concerns regarding steroids, although some severe reactions to azathioprine were also described. 5-ASA therapies did not seem to present any specific concerns in the same way as other medications could: "*Asacol is OK*", "*they've put me on Asacol. They're like a normal tablet*", "*I take the Asacol 3 times a day. If it's bad, I increase it to 4 or 5 times a day, the enemas I keep down because of the cortisone as much as I can ... the codeine phosphate I keep off if I can*", *" I have a feeling the Asacol is like a calming gel or something like that*". Interestingly the fear of surgery or the need for more "serious" drugs could act as a reinforcer for the need to take medication regularly and seek health care at the first sign of a relapse. "*I know how serious it is now you see. At first I didn't, at first I thought it was just a little thing that was passing through and now I know what the other results could be if I delayed. It could be a bag (colostomy) or more drugs more serious drugs*." (male with crohn's disease, 26 years).

A range of other drugs were taken by participants, including analgesics, loperamide, prochlorperazine, betablockers, tranquilisers and antibiotics. Medication management became increasingly more complex when medication for other conditions was necessary. There were concerns that medicines may interact with each other, that the body becomes immune after long-term use and a reluctance to be prescribed further medication. These concerns were not always expressed to the doctor and could be influential in delaying seeking care (see table 4).

**Table 4:** Concerns over long-term and multiple medication use

*"I keep looking at myself and thinking I am on blood pressure tablets, cholesterol tablets, beta-blockers, pain killers for your heart. I take something like nineteen tablets a day and think to myself Jesus, if I go to the doctors to see him about anything else I'll have even more tablets. I don't want more tablets, I want less tablets. And I keep thinking to myself I'll go to the doctors and say "look are you sure these tablets are doing me any good? Isn't one counteracting one which is counteracting another, can't you cut them down?" *(male with ulcerative colitis, 55 years)

*"I've reached saturation level as far as drug therapies are concerned and the consequence of that is that I'm developing other problems*." (male with ulcerative colitis, 65 years)

*"They fight with each other these tablets as they are going down.. I take 2 sleeping tablets; I take my paracetamol, my codeine, my folic acid and my prednisolone. It's a wonder you don't OD really, all these tablets*." (female with crohn's disease, 58 years)

### Perceived impact of actual or potential symptoms

Balancing the concerns of taking medication and the concerns of what might happen without medication was part and parcel of everyday life for those living with IBD. Uncontrolled symptoms could mean pain, embarrassment, faecal incontinence, interference with everyday activities and potential surgery. Just as the acceptance of medication develops over time, concerns over the consequences of living with their illness could also alter with experience, in particular with the severity and frequency of relapses and ongoing symptoms. For many participants, the fear of the impact of actual or potential symptoms is in fine balance with how necessary they perceive their medication to be (see table 5).

**Table 5:** Impact of actual or potential symptoms on attitudes to medication

*"... you can't go anywhere without your tablets, you can't go anywhere without your Imodium you know. You walk around with a spare pair of knickers in your bag and wet wipes and everything just in case. ...I've only ever had one accident (faecal incontinence) and you know it frightened the life out of me." *(female with crohn's disease, 44 years).

*"so, I think if I didn't take them, I am sure I would be poorly." *(female with ulcerative colitis, 56 years)

*" do I keep throwing this amount of drugs into myself which I am sure must be helping me in some point but damaging me in another, or do I risk the ulcerative colitis giving me another year like I had last year, which was absolutely awful, so it's really again you are between a rock and a hard place." (*female with ulcerative colitis, 42 years)

### Willingness to self-manage

Almost two thirds of participants reported they were keen to self-manage their medication. Around a third had a supply of steroids available to take in case of relapse. This saved them "bothering the doctor", waiting unnecessarily for an appointment or having to get to the surgery when fear of faecal incontinence made travel difficult. *"so every time I'm really ill, I take them (supply of steroids at home) .. I don't mind doing that. I mean it's better than keep running to the doctors all the time isn't it? You know I think he'd like me to go more. I'm alright managing it myself to be honest*". (male with ulcerative colitis, 40 years). As mentioned above, medication management became increasingly complex when medication for other conditions was involved. Participants provided detailed accounts of the numbers of tablets they took and when they had to be taken. Most of them were keen to self-manage their medication and many felt confident in increasing or decreasing their daily dosage according to the course of their illness. The inability to self-manage a flare-up or usual symptoms such as pain or bleeding and thus the perceived need for steroids or pain relief were the most important factors in seeking health care. Responsibility for medication taking belonged to the patient, albeit in certain cases after "permission" from the doctor (see table 6).

**Table 6:** Self-management

*"So usually what they tend to do is give me 100 tablets. It is nice in the sense that they trust me that I know how to control it." *(female with ulcerative colitis, 30 years).

"*I would just increase it (medication) myself now... before I used to go (to the doctor) really just to get the go ahead to increase. Well now I don't feel that I have to, so there's a bit more knowledge that's just come with experience." *(female with ulcerative colitis, 56 years).

Although some participants reported satisfactory relationships with their doctor based on a negotiated approach, directly challenging a doctors decision was felt to be inappropriate. "*my doctor is fully supportive of me.. so he tends to say okay what do you want to do with this and I say give me my steroid enemas*." (female with ulcerative colitis, 42 years). *"I feel azathioprine is the drug that gets me right and he (doctor) always prescribes me steroids... you can't say anything, you feel like you should. My consultant gets me right as soon as I am on the azathioprine." *(male with crohn's disease, 30 years). Although the doctor was often seen solely as a "medication provider", the importance of good communication and information is evidenced by the following quote from one female participant from the focus group. "*maybe there was some misinterpretation of what I was supposed to do, but I didn't really feel I had enough knowledge to actually handle my own problems.. I would take the tablets and as soon as the symptoms stopped I just stopped taking the tablets... I wasn't really sure what I was supposed to do and it's been pointed out to me quite recently that I wasn't really using the medication in the way that it was supposed to be used." *(female with ulcerative colitis, 36 years).

## Discussion

When illness specific medications are felt necessary to control symptoms that are significantly impacting on quality of life, more general anti-drug beliefs can be compromised. How individuals deal with this conflict will affect their medicine taking in different ways and may change over time. It is possible, moreover, that patients develop more positive attitudes towards specific medications in order to reduce the conflict between the requirement to take a medication everyday and their negative feelings towards taking drugs. For example, those patients who have negative views of drug taking may, nevertheless, perceive the benefits of their 5-ASA therapy to outweigh any harm due to the necessity of controlling symptoms. To reduce this inconsistency, it is possible that they re-assign more positive specific beliefs to their 5-ASA therapy, such as perceiving it to be a "calming gel" or "normal medicine". This is potentially easier to achieve in the absence of side-effects or through direct experience of its benefits. Although, the fairly positive attitudes to maintenance 5-ASA therapy in contrast to steroids can be explained in terms of a lack of experienced side-effects or by differing routes of administration, negative attitudes to steroids were, nevertheless, less important when their necessity was deemed greater or when they were outweighed by their positive effects on the maintenance of usual activities.

The health care provider is often seen as the "medication provider", whereas many patients see their role as "medication manager", particularly in regards to altering dosages and taking steroids during flare-ups. "Permission" from their doctor to self-manage can help promote positive feelings of control and trust within this role. Sufficient information, however, is required to enable patients to self-manage their medication safely and effectively. Beliefs about medication can inhibit seeking health care when the concerns are high but also promote seeking care when the necessity is high.

### Relation to other studies

The importance of health beliefs in adherence to a treatment regime is well recognised [1,2]. Our findings are compatible with existing theories and models of health behaviour. For example, the balance of concerns and acceptance of the necessity of medication is similar to the concepts of perceived barriers and benefits of action from the health belief model [26] as well Horne and Weinman's research [13] on perceived risks and benefits in medication adherence. The narratives of participants in our research mirrored those reported by Townsend et al [16] on individuals with chronic multiple morbidities in that they "self regulated their drug use in an attempt to gain equilibrium, relief from symptoms or sense of a normal life", and showed a "reluctance to take drugs yet an inability to be free of them". Sewitch et al [7] suggest that health beliefs play a stronger role than quality of life or patient/physician relationship in the variance in medication taking intention, but that those with more severe disease were more likely to consider the costs and the benefits rather than just the costs as is the case with those with less severe disease. Their findings suggest that patients with more experience of IBD may be less likely to deliberately stop taking their medication. It is possible that the participants in our study, who had all been diagnosed for at least two years and had all experienced very low disease related quality of life, may weigh up the benefits and costs of their medication differently to those with a more recent diagnosis, or whose disease related quality of life has not been affected to the same extent. This may be worthy of further research as acceptance of medication and the illness were related and occurred over time. The link between acceptance of illness and medication use has also been described in qualitative research in other chronic diseases which has identified different types of medicine user [15,27]. Narratives from this study suggest that the way individuals use their medication changes over time and with experience of their illness. A quarter of patients with IBD are already treating relapses themselves without guidance and guided self-management can reduce health provision costs and increase confidence in patients' ability to cope with their condition [28-30]. We found that many patients have positive attitudes towards managing their own medication and are often already self-managing. This study, moreover, also highlights the importance of medication for respondents in maintaining a "normal life" and details how a patient's relationship with their health care provider can influence the way in which they manage their medication and, ultimately, their illness. Some evidence, nonetheless, suggests that adherence in GI diseases has shown an upward trend over the last 50 years and is higher than in many other common conditions [4]. It is possible that improvements in treatment options and care provision for IBD have resulted in increased confidence and more positive experiences of maintenance therapy.

### Strengths and weaknesses

As a qualitative study, the usefulness of our findings lies in the rich narratives which allow an in-depth description of the beliefs and attitudes towards medication among patients with IBD. Triangulation of methods and analysis strengthen the validity of the study. Participants were diverse in their demographic characteristics and extent of their disease and included patients not under specialist care. Because of the low prevalence of minority ethnic groups in the study area, only one perspective from a patient of Asian origin was gained and further research would be necessary for comparisons of patients from different cultural backgrounds. Participants were selected from those with the lowest quality of life scores from a previous community based survey of individuals with established IBD [22] to allow for narratives that drew upon a variety of experiences in terms of relapse frequency, disease severity and medicine taking. Rubin et al's findings indicate that these patients are most likely to be under specialist care for IBD and therefore provide a range of views from patients usually accessible to health care providers [22]. Further research would, however, be necessary to identify differences in medication beliefs in patients with better quality of life, with more recent onset of disease, under different care conditions and with different levels of actual adherence.

### Implications for practice

This study provides an in-depth understanding of the concerns and beliefs that patients with IBD have about medication and how these can change over time. Pro-actively eliciting patients' beliefs about specific medications and their attitudes towards their illness can provide useful indicators of potential adherence to their medication regime. In addition, the findings suggest that many IBD patients are keen to self-manage their medication and may already be doing so to varying degrees. Accurate information on potential risks and benefits of medications as well as support, encouragement and feedback are needed to enable patients to do this safely. Managing this process effectively may not only have a positive effect on adherence but could potentially improve the doctor/patient relationship, satisfaction with care, health outcomes and health related quality of life, and result in more efficient use of health care services. Supporting patients effectively to self-manage their condition is not a straightforward task, however, and training in this area may be beneficial [30-34].

## Conclusion

We conclude that, in patients with IBD, perceived necessity of medication is weighed against general and specific concerns as well as outcome expectancies. This process is related to acceptance of their diagnosis and their illness and can therefore change over time, with experience and in some cases with disease activity. The findings are compatible with existing models of health behaviour and research from other chronic diseases. More in-depth understanding of the concerns and beliefs that patients with a relapsing chronic illness such as IBD have about medication and how these change over time could support better guided self-management, and may improve health status.

## Competing interests

The author(s) declare that they have no competing interests.

## Authors' contributions

NJH carried out data collection and analysis and drafted the manuscript. GPR participated in the conception and design of the study and helped to draft the manuscript. APSH participated in the conception and design of the study. AD carried out data collection and analysis. All authors read and approved the final manuscript.

## Pre-publication history

The pre-publication history for this paper can be accessed here:


